# The diagnostic utility of nanopore targeted sequencing in suspected endophthalmitis

**DOI:** 10.1007/s10792-023-02665-7

**Published:** 2023-03-20

**Authors:** Xuejie Li, Ziyue Li, Ming Wang, Aisi Fu, Xinlei Hao, Xinyang Guo, Jiashuang Gu, Wei Jin, Anhuai Yang

**Affiliations:** 1 Eye Center, Renmin Hospital of Wuhan University, Jiefang Road 238#, Wuhan, 430060 Hubei Province China; 2 Department of Clinical Laboratory, Renmin Hospital of Wuhan University, Wuhan, 430060 Hubei Province China; 3 Wuhan Dgensee Clinical Laboratory Co, Ltd. Wuhan 430075, Hubei Province, China

**Keywords:** Endophthalmitis, Nanopore targeted sequencing, Diagnosis, Antibiotics, Intraocular fluid testing

## Abstract

**Purpose:**

This paper aimed to assess the diagnostic utility of a newly developed gene-based technology-nanopore targeted sequencing (NTS) in suspected endophthalmitis patients.

**Methods:**

This retrospective study included 43 patients (44 eyes) with suspected endophthalmitis. NTS was applied along with microbiological culture to detect unknown pathogens in intraocular fluid samples. The diagnostic utility of NTS was mainly evaluated from three aspects, including the positivity rate of bacterial/fungal presence, diagnostic turnaround time and the frequency of change in treatment based on etiology test results. Non-parametric, two-sided Wilcoxon rank sum test, the McNemar’s test and the kappa statistic were used for statistical comparisons.

**Results:**

NTS showed significant advantages over traditional culture in positivity rates and diagnostic time (*P* < 0.001, kappa = 0.082; *Z* = −5.805, *P* < 0. 001). As regards antibiotic strategy, 17 patients (39.53%) and 5 patients (11.63%) underwent medication change following NTS and culture results respectively (*P* < 0.001, kappa = 0.335). With reasonable use of antibiotic and surgical intervention, most patients responded favorably, judged by significantly improved visual acuity (*Z* = −4.249, *P* < 0.001). The mean duration of hospitalization was 8.49 ± 2.45 days (range, 1–16 days).

**Conclusion:**

The high efficiency feature of NTS in pathogen detection renders it a valuable supplementary to traditional culture. Additionally, it has facilitated patients’ management for the early and precise diagnosis of endophthalmitis.

## Introduction

Endophthalmitis is one of the most devastating ocular diseases, and it can lead to permanent vision loss within a few days. Most cases of exogenous endophthalmitis are caused by infectious pathogens introduced to the eye from either the ocular surface or the environment (such as keratitis or trauma). Endogenous endophthalmitis (EE) cases are rare but highly destructive, and it usually result from chronic systemic infections (such as liver abscesses, endocarditis, or urinary tract infections [1]). Due to the delicate anatomy of the eye and the limited ocular sample size, it has presented a diagnostic challenge to identify the diversified causative organisms in endophthalmitis cases. Prognosis may be poor owing to delayed diagnosis and lack of targeted anti-microbial treatment. Therefore, the most critical issue in diagnosing endophthalmitis is the identification of clinical microbiology, as early identification of causative pathogen(s) is imperative to guide further anti-microbial treatment.

Although the positivity rate is only about 38%-64%, culture performed in vitreous humor (VH) or aqueous humor (AH) samples remains the gold standard for the pathogen detection in endophthalmitis patients. Nevertheless, the time required for different pathogen culture ranges from a few days to several weeks, often resulting in delayed diagnosis [2]. Thus, molecular diagnostic techniques emerged. Multiplex PCR testing have the potential to identify pathogens rapidly, especially in culture-negative cases. However, this method requires primers based on prior assumptions about the species and only detects a narrow array of pathogens. Next-generation sequencing (NGS) technologies have contributed to a broad detection range and it boasts a high accuracy for laboratory diagnosis of endophthalmitis. More recently, nanopore-targeted sequencing (NTS) came to light and a set of targeted microbial tags were incorporated [3]. With targeted gene amplification, the unique advantage of its long-read and real-time analysis can be achieved.

In this article, we further verified the effectiveness of NTS by focusing on clinical-oriented aspects. The purpose of our study was to assess the diagnostic utility of NTS in suspected endophthalmitis cases and explore the role of NTS intervention in the early clinical decisions.

## Materials and methods

### Study design and patients

By reviewing the medical records from the hospital information system (HIS) of the Ophthalmic Center, a retrospective study was performed in the endophthalmitis patients who underwent NTS and culture simultaneously between January 1, 2018, and July 24, 2022. This study was carried out following the institutional guidelines and ethical standards of the 1964 Declaration of Helsinki and was approved by the Institutional Review Board of Renmin Hospital of Wuhan University (WDRY2019-K056). All patients provided their written informed consents to participate in this study.

The criteria included: 1. Recent history of eye surgery or penetrating ocular trauma, or with other predisposing factors; 2. Typical manifestations of significant loss of vision, ocular pain, ocular redness, etc.; 3. Marked intraocular inflammation like hypopyon and vitritis on ocular examination; 4. Intraocular fluid including aqueous humor (AH) or vitreous humor (VH) was collected and sent for NTS and microbiological culture simultaneously; 5. With a minimum 3-month follow-up.

The patients’ records were reviewed including demographic characteristics, best corrected visual acuity (BCVA) at their first visit, clinical features on slit-lamp examination, disease course and treatment, etiology test results (NTS and/or culture results), the time to confirmatory diagnosis, management details, length of hospitalization and BCVA at last follow-up.

### Outcome measures

The positivity rate of bacterial/fungal presence, diagnostic turnaround time, and frequency of changes in treatment protocol based on etiology test results (defined as any change directly attributable to sequencing or culture results) are used as primary outcome measures. Continuous data were presented as mean and standard deviation, whereas categorical data were presented as the number of suffered eyes and percentage. For statistical analysis, vision was reported as mean and median logMAR vision with Snellen conversion. Non-Snellen acuities were recorded in the following fashion: a visual acuity of 2/800 on Feinbloom’s low vision chart was considered equivalent to counting fingers (CF), and it was defined as 2.6 logMAR. Likewise, we used logMAR values of 2.7, 2.8, and 2.9 to represent the vision of hand movement (HM), light perception (LP), and no light perception (NLP), respectively.

### Sample collection

To limit contamination, intraocular fluid (AH or VH) samples for NTS were obtained under strictly sterile conditions. AH samples were obtained through anterior chamber paracentesis, and VH samples were obtained during the biosurgery procedure. The operations were performed by the same operator, and purulent lesions and inflammatory exudate were cleared as much as possible during the operations. All clinical specimens were then sent to the clinical laboratory with specific pretreatment. The experimental procedures were performed by well-trained laboratory technicians in a qualified laboratory (Wuhan Dgensee Clinical Laboratory Co., Ltd. Wuhan 430,075, China).

### Culture method

Gram stain and KOH mount were routinely performed on aqueous humor or vitreous specimens. The remaining samples were inoculated on Columbia blood AGAR basal medium (for bacteria) and Sabouraud glucose AGAR medium (for fungi) using a BACTEC 9120 culture system (BD Diagnostics, Sparks, MD). For culture-positive cases, isolated fungi and/or bacteria were identified using the Vitek 2 Compact automated identification system (bioMerieux, Marcy L 'Etoile, Huang et al. 1061 France) and MALDI Biotyper mass spectrometry (Bruker, Marcy L 'Etoile, Huang et al. Madison, WI).

### Sequencing method

#### Preprocessing and DNA extraction

Intraocular fluid samples were centrifuged at 20,000 ×*g* for 10 min. The supernatant was removed, and 200 μL of the specimen was reserved for DNA extraction (Sansure DNA Extraction Kit, Changsha, China). All primers used in this study have been described in a previously published article [, 3, 4].

#### NTS library construction and sequencing

Amplification of the bacterial 16S rRNA gene was performed in a 20 μL reaction system with 8 μL of extracted DNA, 2 μL of barcoded primer (10 μM), and 10 μL of 2 × KOD TM PCR Master Mix (TOYOBO) using the following cycle: 98 °C for 3 min; followed by 35 cycles at 98 °C for 10 s, 55 °C for 5 s, and 68 °C for 10 s; and a final elongation step at 68 °C for 5 min.

Amplification of the fungal internal transcribed spacers 1 and 2 (ITS1/2) was performed in the same reaction system and the primer mix without the barcode was used in the PCR procedure. The PCR product was purified with 0.8 × AMpure beads (Beckman Coulter) and eluted in 10 μL Tris–EDTA (TE) buffer. Then, 5 μL of the eluate was used for PCR with 5 μL of the barcoded ITS1/2 primer set (10 μM), and 10 μL 2 × Phusion U Multiplex PCR Master Mix. The cycle was as follows: 98 °C for 3 min; followed by 10 cycles at 98 °C for 10 s, 55 °C for 5 s, and 68 °C for 5 s; and a final elongation step at 68 °C for 5 min.

Barcoded products of 16S rRNA ITS1/2 gene amplification from the same samples were pooled in a mass ratio of 10:3. Pooled products from the different samples were mixed equally and 1D ligation kits (SQK-LSK109; Oxford Nanopore) were used to construct sequencing libraries. Then, the library was sequenced using Oxford Nanopore MinION. TE buffer was run in each batch as a negative control throughout DNA extraction, target amplification, library construction and sequencing.

#### Bioinformatics analysis

Fast5 files generated by MinION were real-time base called and demultiplexed using Albacore v2.3.1. Low-quality reads (less than 7) were filtered. Porechop was used to trim the barcodes and adapters from the raw reads. Afterwards, the filtered sequencing reads were mapped to the reference databases downloaded from the 16S rDNA/ITS reference database maintained by NCBI FTP (ftp://ftp.ncbi.nlm.nih.gov/refseq/TargetedLoci) using Blast, and the taxonomy of each read was assigned according to the taxonomic information of the mapped subject sequence.

### Statistical analysis

IBM SPSS Statistics Software Version 20 (SPSS, Inc, Chicago, Illinois, USA) was used for data analysis. Continuous variables like VA and diagnostic time were compared using a non-parametric, two-sided Wilcoxon rank sum test. The McNemar's test and the kappa statistic were used to compare the diagnostic positivity rates, the frequency of polymicrobial infection, and the frequency of treatment change between two methods. P value less than 0.05 was considered to be statistically significant.

## Results

### Basic information and clinical features of the patients

Demographic characteristics, disease course, clinical features, management details, and visual outcomes were demonstrated in Table 1. A total of 30 males and 13 females were involved in our study and the mean age was 54.86 ± 18.29 years. Most cases (23/43, 53.49%) occurred after penetrating ocular injuries from metal objects, sticks, pencils, or stones. 16 patients (16/43, 37.21%) developed severe ocular inflammation after ophthalmic surgeries (14 after cataract surgery, 1 after pterygium excision, and 1 after implantable collamer lens (ICL) implantation). 1 patient had chronic comorbid conditions of liver abscesses and was suspected of binocular endogenous endophthalmitis. The mean interval between the insult (surgery/ trauma/ infection) and manifestation of the injury was 7.26 ± 7.79 (range, 1–30 days).

**Table 1:** Clinical and demographic details of the patients with presumed infectious endophthalmitis included in the study

Sample ID	Age	Sex	Cause (interval between diagnosis and event, days)	Presenting VA	Clinical features	Surgery	Treatment	Final VA	NTS result	Culture result	Length of hospital stay
1	63	M	Injury-stone (1)	NLP	Fbrinous exudation	Phaco + PPV + SOT + IVT	V + C + Dx	NLP	+	–	12
2	62	M	Injury-iron wire (3)	LP	Hypopyon	PPV + SOT + IVT	V + C + intravenous and intraocular VCZ	NLP	+	+	11
3	70	F	Surgery-pterygium excision (15)	LP	Fibrinous exudation	IVT	V + C	HM	+	–	12
4	36	M	Liver abscess (15)	FC/0.8	KP(+)	Phaco + PPV + SOT + IVT	IPM + MEM + ETM + CIP + CLR + MH	0.1/1.0	+	–	8
5	70	M	Surgery-Phaco (2)	LP	Hypopyon	PPL + PPV + SOT + IVT	V + C	FC	+	–	5
6	25	M	Surgery-ICL (3)	HM	Fibrinous exudation	AC wash + ILE + IVT	V + C + Dx	0.12	–	–	4
7	73	M	Surgery-Phaco (5)	FC	Hypopyon	ILE + PPV + SOT + IVT	V + C	0.03	+	–	6
8	69	F	Injury-iron wire (7)	LP	Hypopyon	Eye removal	V + C + intravenous CIP	–	+	+	8
9	50	M	Injury-metal (1)	HM	KP(-)	PPL + PPV + SOT + IOFB-R + IVT	V + C	HM	+	+	9
10	51	M	Injury-iron wire (3)	HM	Hypopyon	PPV + SOT + IVT	V + C	0.05	+	+	7
11	41	M	Injury-stick (7)	FC	KP(-)	PPV + IVT	V + C	FC	+	–	8
12	54	M	Injury-steel nail (3)	HM	Hypopyon	PPL + PPV + SOT + IVT	V + C + intravenous LEV	0.01	+	–	7
13	68	M	Injury-nail (1)	LP	Hypopyon	CTR + Phaco + IOFB-R + PPV + SOT + IVT	V + C	FC	+	–	10
14	36	M	Injury-iron scrap (28)	HM	Fibrinous exudation	AC tap + IVT	V + C	0.04	+	–	5
15	67	M	Surgery-Phaco (4)	HM	KP(+ +)	AC wash + PPV + SOT + IVT	V + C	0.12	–	–	10
16	7	F	Injury-pencil (1)	0.1	Fibrinous exudation	AC wash + IVT	V + C	0.25	+	–	11
17	50	M	Injury-nail (1)	LP	Fibrinous exudation	Phaco + PPV + SOT + IVT	V + C + Dx	FC	–	–	7
18	55	M	Injury-iron wire (15)	0.05	Hypopyon	PPV + SOT + IVT	V + C	FC	+	–	9
19	54	M	Injury-nail (30)	FC	KP(-)	PPV + SOT + IVT	V + C	0.1	+	–	12
20	55	M	Surgery-Phaco (6)	HM	Fibrinous exudation	ILE + PPV + SOT + IVT	V + C	FC	+	–	6
21	58	F	Surgery-Phaco (7)	FC	KP(-)	PPV + IVT	V + C	0.2	–	–	7
22	50	M	Injury-iron wire (2)	0.2	Blood	CTR + PPV + SOT + IVT	V + C	0.25	–	–	9
23	51	F	Surgery-Phaco (6)	HM	KP(+ +)	AC wash + PPV + SOT + IVT	V + C	FC	+	–	6
24	70	M	Surgery-Phaco (15)	FC	Fibrinous exudation	PPV + SOT + IVT	V + C + intravenous CIP	0.2	+	–	5
25	58	M	Surgery-Phaco (30)	HM	Hypopyon	AC wash + PPV + SOT + IVT	V + C	0.08	+	–	8
26	54	M	Injury-stone (3)	HM	Fibrinous exudation	PPV + SOT + IVT	V + C	FC	+	–	7
27	58	M	Surgery-Phaco (9)	HM	Fibrinous exudation	AC wash + IVT	V + C + Dx	0.08	–	–	13
28	80	F	Surgery-Phaco (2)	LP	Hypopyon	ILE + PPV + SOT + IVT	V + C + intravenous LEV	HM	+	+	10
29	48	F	Injury-nail (2)	FC	Hypopyon	PPV + SOT + IVT	V + C + intravenous LEV	FC	+	–	9
30	58	F	Injury-bone fragments (2)	HM	Hypopyon	AC wash + IVT	V + C	FC	+	+	16
31	64	M	Injury-metal (4)	HM	Hypopyon	AC wash + IVT + eye removal	V + CIP + intravenous and intraocular VCZ	–	+	+	11
33	51	M	Keratitis (4)	LP	Hypopyon	PPV + SOT + IVT	V + C + intravenous LEV	HM	+	–	9
34	58	M	Surgery-Phaco (12)	FC	Fibrinous exudation	AC wash + IVT	V + C + intravenous and intraocular VCZ	0.15	+	–	8
35	70	M	Surgery-Phaco (15)	FC	Hypopyon	PPV + SOT + IVT	V + intravenous and intraocular VCZ	0.06	+	–	10
36	94	M	Keratitis (15)	HM	Hypopyon	AC wash + IVT	V + C + intravenous ETM	FC	+	–	5
37	64	F	Surgery-Phaco (4)	FC	KP (+), fibrinous exudation	PPV + IVT	V + C + intraocular Gen	0.4	+	–	8
38	41	M	Injury-iron wire (1)	HM	Hypopyon, fibrinous exudation	Phaco + PPV + SOT + IVT	V + C + intravenous Au + ETM + VCZ and intraocular Gen	LP	+	–	8
39	57	F	Keratitis (15)	HM	KP (+)	PPV + SOT + IVT	V + C + intravenous ETM	FC	+	–	8
40	7	F	Injury-metal (1)	HM	Fibrinous exudation	CTR + ILE + PPV + SOT + IVT	V + C + intravenous Au	HM	+	–	8
41	80	M	Surgery-Phaco (3)	HM	Hypopyon	PPV + ILE + SOT + IVT	V + C + intravenous LEV	HM	+	+	6
42	4	F	Injury-plant (2)	LP	Hypopyon	CTR + AC wash + ILE + PPV + SOT + IVT	V + C	HM	+	+	9

Demographic characteristics, disease course, clinical features, management details and visual outcomes were demonstrated in Table 1. *M*: male; *F*: female; *Phaco*: phacoemulsification; *IOL*: Intraocular lens implantation; *ILE*: Intraocular lens extraction; *CTR*: Corneal tear repair; *IOFB-R*: Intraocular foreign body removal; *V*: Vancomycin; *C*: Ceftazidime; *LEV*: levofloxacin; *Dx*: Dexamethasone; *Gen*: Gentamicin; *AC Tap*: Aqueous biopsy. *IPM*: imipenem; *MEM*: meropenem; *ETM*: etimicin; *CIP*: ciprofloxacin; *CLR*: clarithromycin; *MH*: minocycline hydrochloride; *MFLX*: moxifloxacin hydrochloride; *Au*: amoxicillin/clavulanate potassium.  +  : NTS/culture positive;  − : NTS/culture negative

**Fig. 1:**
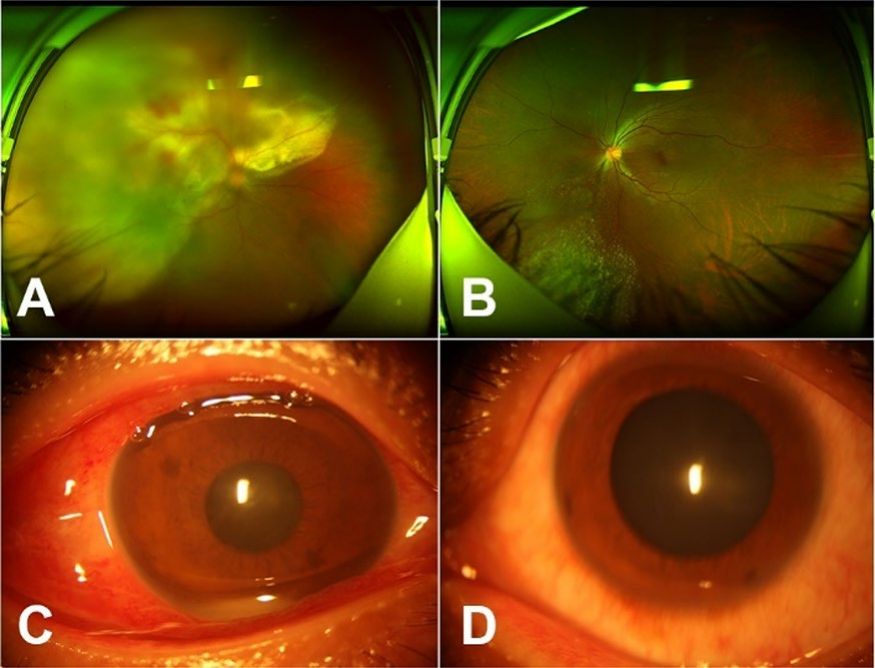
Anterior chamber and bilateral fundus photographs in patient #4. **a** Superotemporal large gray-white lesion and subretinal abscess in the right eye. **b** Inferonasal miliary grain lesion in the left eye. **c** and **d** Inflammatory exudation in the anterior chamber of the right eye and the left eye

Presenting visual acuity (VA) results were as follows: no light perception (NLP; *n* = 2/44, 4.55%), light perception (LP; *n* = 9/44, 20.45%), hand motions (HM; *n* = 19/44, 43.18%), counting fingers (10/44, 22.73%), and undetermined VA (*n* = 4/44, 9.09%). These cases were characterized by severe anterior chamber inflammation and dense infiltration in the vitreous cavity. Hypopyon inflammatory/fibrinous exudation was observed in 33 of 44 eyes (75%). Visualization in the posterior segment view was poor in all 44 eyes due to severe vitritis (Fig. 1).

The visual outcome was defined as improvement, stabilization, and deterioration. Improved visual outcomes were found in 31 eyes (70.45%), and stabilized visual outcomes were found in 7 eyes (15.91%). 6 eyes (13.64%) had worse or deteriorated visual outcomes. 3 patients whose initial visions were LP, HM, and NLP respectively, underwent eye removal for severe inflammation involving the whole eye. Overall, the VA at the last follow-up was significantly improved compared with VA at the first visit (*Z* = −4.249, *P* < 0.001) (Table 2).

**Table 2:** Summary of the diagnostic utility of NTS as well as culture, and the final visual acuity of 43 patients (44 eyes) involved

Diagnostic utility	NTS	Culture	*P* value
Positivity rate	86.05% (37/43)	20.93% (9/43)	< 0.001 kappa = 0.082
Polybacterial result	22/43 (51.16%)	1/43 (2.33%)	< 0.001 kappa = 0.044
Turnaround time, days	1.23 ± 0.43	3.58 ± 0.88	< 0. 001 *Z* = −5.805
Therapeutic strategies changed	17/43 (39.53%)	5/43 (11.63%)	< 0.001 kappa = 0.335
Final visual acuity	Number of patients		
Improvement	31		
Stabilization	7		
Deterioration	6		

### Clinical microbiology detected by NTS and culture

The results for NTS and culture are shown in Tables 3 and 4. In total, the positivity rate of NTS was 86.05% (37/43), and that of culture was 20.93% (9/43) (*P* < 0.001, kappa = 0.082). In all 9 culture-positive samples, the major pathogens were highly consistent with NTS results. Of the 37 NTS-positive samples, 22 showed polymicrobial infection. Whereas of the 9 culture-positive samples, only 1 showed polymicrobial infection. The frequency of polymicrobial results between NTS (22/43, 51.16%) and culture (1/43, 2.33%) was statistically significant (*P* < 0.001, kappa = 0.044).

**Table 3:** NTS species-specific reads and taxonomic lineage of culture-positive samples

Sample ID	Gram stain/KOH mount/culture result	Taxonomic Lineage by NTS	N. of read
2	*Enterobacter kobei, Candida albicans*	*Enterobacter kobei*	92
		*Staphylococcus aureus*	52
		*Candida albicans*	8934
8	*Enterobacter ludwigii*	*Enterobacter ludwigii*	160
9	*Acinetobacter junii*	*Haemophilus parainfluenzae*	11,866
		*Acinetobacter junii*	1300
10	*Staphylococcus epidermidis*	*Staphylococcus epidermidis*	1302
28	*Enterococci faecalis*	*Enterococci faecalis*	47,620
30	*Pasteurella multocida*	*Pasteurella multocida*	5902
		Sac fungi*	11,424
31	*Colletotrichum spp.*	*Comamonas testosteron*	470
		*Colletotrichum spp*	17,293
41	*Enterococci faecalis*	*Enterococci faecalis*	116,083
42	*Streptococcus pneumoniae*	*Streptococcus pneumoniae*	20,138

**Table 4:** NTS species-specific reads and taxonomic lineage of bacterial and fungal culture-negative samples

Sample ID	Taxonomic lineage	N. of read
1	*Sphingomonas paucimobilis, Staphylococcus aureus*	50, 15
3	Dialister spp.*, Cladosporium halotolerans*	23, 24
4	*Stenotrophomonas maltophilia (R)*	932
	*Mycobacterium abscessus, Stenotrophomonas maltophilia (L)*	147, 31
5	*Streptococcus sanguinis*	155
7	Streptococcus gordonii, Paraburkholderia dipogonis*	900, 320
11	*Staphylococcus epidermidis*	15
12	Haemophilus influenzae, Yarrowia lipolytica*	12, 21
13	*Streptococcus midis, Achromobacter spp.*	420, 137
14	Sporidiobolus spp. *	4688
16	*Streptococcus pneumoniae*	2489
18	Micrococcus kristinae*	35
19	Anaerococcus prevotii*	32
20	*Staphylococcus epidermidis*	9595
23	Streptococcus gordonii, Enterobacter cancerogenus*, Meyerozyma guilliermondii*	67, 54, 2633
24	Staphylococcus saccharolylicus, Lactococcus spp.*, Yarrowia lipolytica*	1114, 757, 13,925
25	Corynebacterium tuberculostearicum*	1178
26	Atopobium parvulum*, Streptococcus parasanguis, Meyerozyma guilliermondii*	259, 173, 11,140
29	*Aeromonas caviae, Morganella morganii*	30,153, 242
32	*Klebsiella pneumoniae, Enterobacter asburiae*	1060, 884
33	*Escherichia coli*	507
34	Corynebacterium jeikeium*, Aspergillus gracilis	163, 1170
35	*Staphylococcus aureus, Aspergillus penicillioides*	109, 3232
36	Porphyromonas bennonis*	346
37	Finegoldia magna*, Staphylococcus aureus, Moraxella osloensis, Corynebacterium confusum*	947, 61, 2232, 311
38	*Haemophilus influenzae, Candida parapsilosis*	24,246, 21,672
39	Staphylococcus aureus, Anaerococcus nagyae*	54,114, 49
40	Bacillus cereus, Clostridium perfringens, Eubacterium tenue*	45,428, 2858, 1951
43	Aeromonas veronii, Citrobacter freundii, Lactococcus lactis*	52457, 257, 791

^*^Reads represent the number of sequences of the microorganism detected at the genus or species level. The organism considered as nonpathogenic is marked with an asterisk. To clarify, we have conducted a literature search in Pubmed Database on all the organisms detected. It should be noted that the criteria for classifying pathogenic and nonpathogenic organisms were the epidemiology of the pathogen and the presence of previous cases of associated endophthalmitis

Among 37 patients (38 eyes), a total of 45 species of bacteria and 11 species of fungi were identified by NTS, and no pathogens were detected in 6 eyes. These organisms were divided into pathogenic and non-pathogenic organisms. We have marked non-pathogenic organisms with an asterisk in Tables 3 and 4. The most frequently detected pathogens were *Streptococcus spp.*, followed by *Staphylococcus spp.* and *Enterobacter spp*. Apart from well-recognized causative agents of endophthalmitis mentioned above, unusual and virulent pathogens were also revealed by NTS, including *Sphingomonas paucimobilis, Mycobacterium abscessus, Stenotrophomonas maltophilia, Achromobacter spp., Aeromonas caviae, Morganella morganii, Acinetobacter junii, Colletotrichum spp.*, *Moraxella osloensis*, *Bacillus cereus, Clostridium perfringens, Aeromonas veronii,* and* Citrobacter freundii.*

A total of 20 microorganisms detected by NTS were identified as non-pathogenic. To our knowledge, some organisms have not been detected in any other clinical specimens: *Cladosporium halotolerans, Paraburkholderia dipogonis, Yarrowia lipolytica*, *Sporidiobolus spp., Meyerozyma guilliermondii*, *Lactococcus spp.,* and *Sac fungi*. Whereas some were reported to colonize the oral cavity or the skin, and there were no reports of associated ocular infections caused by these organisms: *Dialister spp., Micrococcus kristinae*, *Anaerococcus prevotii*, *Enterobacter cancerogenus, Corynebacterium tuberculostearicum, Atopobium parvulum, Corynebacterium jeikeium, Porphyromonas bennonis, Finegoldia magna, Corynebacterium confusum, Anaerococcus nagyae, Eubacterium tenue,* and *Lactococcus lactis.*

All the patients obtained NTS results and determined treatment strategies within 1 or 2 days, with an average duration of 1.23 ± 0.43 days. The turnaround time for sequencing in the laboratory was around 8 h. The traditional culture required 3–4 days for bacterial detection and 5–7 days for fungal detection after collection of the specimens. The average turnaround time for culture was 3.58 ± 0.88 days. Thus, there was a significant difference between the two methods in terms of diagnostic time (*Z* = −5.805, *P* < 0.001). In this study, the mean duration of hospitalization in the Ophthalmic Center was 8.49 ± 2.45 days (range, from 1 to 16 days).

### Management and changes in antibiotic strategy by the intervention of NTS

Due to the severity of the endophthalmitis, pars plana vitrectomy (PPV) was needed in 32 cases (74.42%). Each of these patients was first treated by a standard protocol with systemic and topical antibiotics, including intravenous and intravitreal ceftazidime (CEF)/vancomycin (VAN). After receiving NTS reports, 17 of 43 patients (39.53%) changed their antibiotic strategy. In contrast, only 5 of 43 patients (11.63%) were advised to change their medication after obtaining culture results. A significant difference existed in the medical guidance between the two methods (*P* < 0.001, kappa = 0.335).

Specifically, in patients #2, #31, #34, #35 and #38, virulent fungi including *Aspergillus gracilis, Aspergillus penicillioides, Candida albicans, Colletotrichum spp.*, and *Candida parapsilosis* were detected. Then they received additional intravenous and intraocular voriconazole (VCZ)/gentamicin (Gen) immediately. For patient #8, in whom an emerging multidrug-resistant gram-negative bacilli *Enterobacter ludwigii* was detected, levofloxacin (LEV) was intravenously administered instead. In addition, high abundance of *Aeromonas caviae* was found in patient #29. This is a rare and destructive gram-negative bacterium that often lives in sewage and seawater. This patient worked for leech farming and lived in a humid environment, which confirmed the source of the pathogen. Therefore, we added CIP to her systemic antibiotics. The management was similar in patients #12, #24, #28, #33, #37, #39, #40, #41, and #43, and the details of changes in antibiotic strategies were shown in Table 5. Most of these patients responded favorably, but 3 patients underwent enucleation because of severe inflammation involving the whole eye.

**Table 5:** The details of change in antibiotic strategies attributable to NTS

Patient ID	NTS result	Culture result	Initial antibiotics	Change in antibiotic strategies	Therapeutic Effect
2	*Enterobacter kobei, Staphylococcus aureus, Candida albicans*	*Enterobacter kobei* *Candida albicans*	V+C	V +C+intravenous and intraocular VCZ	deterioration
4	*Mycobacterium abscessus, Stenotrophomonas maltophilia*	negative	V+C+ CEM +IPM	IPM+MEM+ ETM + CIP + CLR + MH	improvement
8	*Enterobacter ludwigii*	*Enterobacter ludwigii*	V+C	V+C+intravenous CIP	eye removal
12	Haemophilus influenzae, Yarrowia lipolytica*	negative	V+C	V+C +intravenous LEV	improvement
24	*Klebsiella pneumoniae, Enterobacter asburiae*	negative	V+C	V+C+ intravenous CIP	improvement
28	*Enterococci faecalis*	*Enterococci faecalis*	V+C	V+C+ intravenous LEV	improvement
29	*Aeromonas caviae, Morganella morganii*	negative	V+C	V+C+ intravenous CIP	stabilization
31	*Comamonas testosteroni, Colletotrichum spp.*	*Colletotrichum spp.*	V+C	V +IP+ intravenous and intraocular VCZ	eye removal
33	*Escherichia coli*	*negative*	V+C	V+C+intravenous LEV	improvement
34	*Corynebacterium jeikeium, Aspergillus gracilis*	negative	V+C	intravenous and intraocular VCZ	improvement
35	*Staphylococcus aureus, Aspergillus penicillioides*	negative	V+C	V+intravenous and intraocular VCZ	improvement
37	*Finegoldia magna, Staphylococcus aureus, Moraxella osloensis, Corynebacterium confusum*	negative	V+C	V+C+intraocular Gen	improvement
38	*Haemophilus influenzae, Candida parapsilosis*	negative	V+C	V+C+intravenous Au + ETM + VCZ and intraocular Gen	deterioration
39	*Staphylococcus aureus, Anaerococcus spp.*	negative	V+C	V+C+intravenous ETM	improvement
40	*Bacillus cereus, Clostridium perfringens, Eubacterium tenue*	negative	V+C	V+C+ intravenous Au	stabilization
41	*Enterococci faecalis*	*Enterococci faecalis*	V+C	V+C+intravenous LEV	stabilization
43	*Aeromonas veronii, Citrobacter freundii, Lactococcus lactis*	negative	V+C	V+C+intravenous MFLX	stabilization

### NTS in endogenous endophthalmitis

**Fig. 2:**
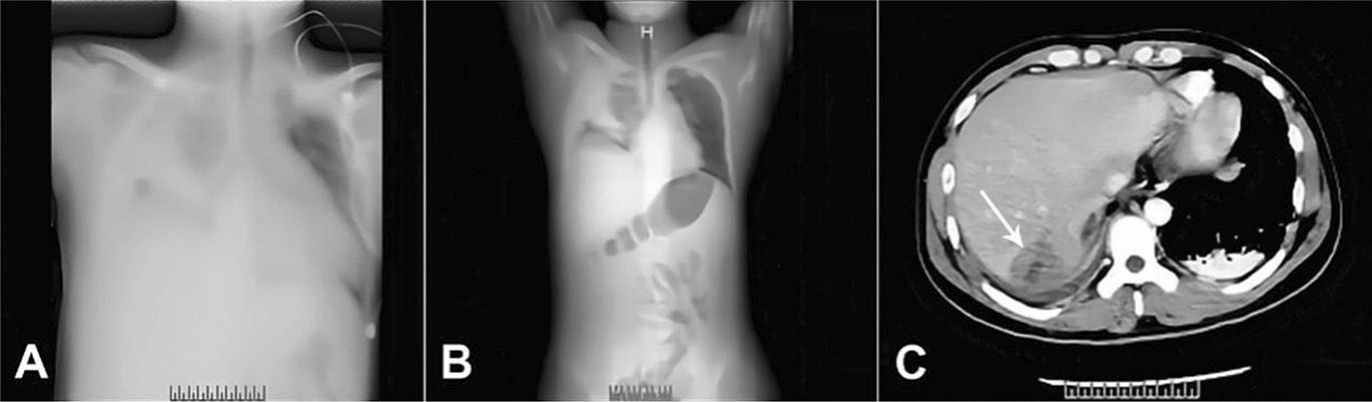
Chest X-ray and computed tomography images of patient #4. **a** Infected lesions in both lungs with bilateral pleural effusion, partially encapsulated. Inadequate expansion of lung tissue at the fluid surface and solid lung changes near the fluid surface. **b** Marked dilatation of the small intestine and acute intestinal obstruction due to inflammatory irritation. **c** Liver abscess (Indicated by a white arrow)

Patient #4 with EE was presented with liver abscess, high fever and sepsis. Later he developed eye pains and vision loss in both eyes (Fig. 2). He was treated with intravenous MEM + VAN and binocular intraocular CEF + IPM, but no sign of symptom improvement was observed in the right eye. Then, Phaco + PPV + SOT + IVT was performed in his right eye to remove the inflammatory lesions. AH samples was collected in both eyes for NTS and microbiological culture. 24 h later, NTS revealed *Stenotrophomonas maltophilia* in the right eye, and *Mycobacterium abscessus* + *Stenotrophomonas maltophilia* in the left eye. Culture of AH samples revealed no pathogen. Subsequent cultures of liver abscesses also reported the presence of *Klebsiella pneumoniae* + *Stenotrophomonas maltophilia* + *Mycobacterium abscessus*, confirming the diagnosis of EE.

In view of the severe condition and the newly detected pathogens by NTS, we switched the systemic antibiotic regimen to “MEM + ETM + CIP + CLR + MH”. Meanwhile, puncture and drainage of liver abscess was performed. Surprisingly, both systemic and ocular symptoms were alleviated considerably. His final BCVA maintained 20/200 in the right eye and 20/20 in the left eye.

## Discussion

### The unique advantages of NTS

Since the ONT first released the MinION to early users in 2014, many proof-of-concept studies have demonstrated its applications in infectious disease diagnostics [5]. For instance, the surveillance of emerging infectious diseases outbreak [6], identification of pathogen drug resistance [, , 5, 7, 8], and disease-related microbial community characterization [9]. Other studies have also provided clinical examples of the validation of NTS for pathogen identification in various samples, including aqueous humor or vitreous fluid [10], blood [11], and nasopharyngeal swabs [12]. In the field of ophthalmology, NTS is ideal for the analysis of microorganisms in AH or VH since a very limited sample volume (0.2 mL) is sufficient for detection [10].

As a third-generation sequencing technology, NTS has two unique advantages over NGS. First, it exhibits higher species-level resolution through a long-read sequencing strategy, which enhances accuracy by avoiding mis-assembly of genomic repeat regions. Second, nanopore-based technology is considered real-time as the data are generated read by read, whereas NGS results are not available until the end of the sequencing run. NTS has the potential to detect microorganisms within minutes of starting sequencing [13] and provide reliable results within 6 h of sample receipt [14]. Therefore, it is particularly useful for early antibiotic administration through timely detection of pathogens.

### Microbiology diagnostics by NTS

Similar to the study by Huang et al. [10], we were able to detect pathogens in intraocular fluid in a very short period of time with a high positivity rate. We also collated the culture and sequencing results of clinically suspected endophthalmitis from other researches in Table 6 (see Appendix A for full trans). Generally, there was a good correlation between NTS and standard culture results in double-positive cases. In this study, we also found that the main organisms identified by both methods were identical. However, the accuracy and sensitivity of the microbial profiles in culture results were poor. One potential explanation was frequent exposures to antibiotics before sample collection, which may have influenced bacterial cultivation. Also due to mutual inhibition mechanisms of bacteria, culture results often reported a single pathogen, indicating the risk of under-detection.

NTS technology has tremendous advantages in detecting multiple infections, especially in the case of mixed bacterial and fungal infections. In this article, polymicrobial results were reported in more than half of the patients by NTS. It allowed early identification of the uncultured and time-consuming microorganisms (e.g. anaerobes and fungi), regardless of prior use of broad-spectrum antibiotics [, 15, 16]. Therefore, NTS may function as a valuable supplementary to diagnostics when culture-based methods are flagged as negative.

### NTS enables early targeted therapy

Optimal clinical decision-making depends on identifying clinically relevant organisms present in the sample. However, conventional culture methods are always too slow and often fail to identify unusual or fastidious organisms. The average waiting times for the results of bacterial and fungal cultures were 48 and 72 h respectively, which is not conducive to guiding targeted antimicrobial therapy, especially for ocular emergencies like endophthalmitis.

While in the case of NTS, even with atypical and low-abundance pathogens, the turnaround time from sample to result was no more than 24 h [, 17, 18]. Thus, NTS enables early targeted therapy by reducing detection time and clinical turnaround time. When atypical and virulent pathogens are detected and inadequate therapy is given, NTS may save vision and reduce the risk of blindness by altering antibiotic therapy without delay [, 15, 16]. When no pathogens are detected or the detected microorganisms are determined to be non-pathogenic, this approach may contribute to an early de-escalation of broad-spectrum therapy, delaying antimicrobial resistance (AMR) [19]. Noticeably, as with the EE case in our study, NTS may be instructive in both topical and systemic medication.

Due to the presence of the blood-retinal barrier (BRB), NTS-guided antibiotic therapy is an effective complementary to patient management, but not a substitute for surgical treatment when persistent vitritis occurs [20]. We noticed the persistent symptoms and poor VA outcomes despite the coverage of broad-spectrum antibiotics in some patients, and PPV was needed to remove purulent lesions. This may be explained by the fact that visual outcomes in endophthalmitis are related to several factors, including presenting visual acuity, the presenting interval, and the promptness of appropriate therapy. Thus, further studies are required to clarify the role of NTS in altering the course of the disease and improving long-term VA. However, it has been noted in an array of literature that although the role of surgical and medical treatment in endophthalmitis varies, the most important intervention remains immediate intravitreal antibiotic injection [21]. Meanwhile, considering that poor visual prognosis of endophthalmitis is strongly associated with the type of pathogens involved, the identification of causative pathogens may still have important implications in predicting visual prognosis in the early stage of the disease.

### Data interpretations for NTS in a clinical setting

NTS is a hypothesis-free approach and it has the potential to detect any unknown DNA-based microorganism in a clinical sample. This not only offers the promise of improved detection of traditional organisms, but also the ability to identify organisms not previously associated with endophthalmitis.

Comprehensive description of the microbial constituents may provide additional benefits: For one thing, multiple pathogens including less-common ones are assessed simultaneously during the initial sequencing run, thereby avoiding many rounds of testing. For another, it allows in-depth investigations of the ocular microbial community. This is vital both in maintaining ocular homeostasis [22] and in the pathophysiology of the disease. Changes in the eye microbiome have been confirmed to be linked with disease states like dry eye, diabetic retinopathy, glaucoma, macular degeneration, and infectious keratitis [23]. In recent studies, nanopore sequencing was proposed to monitor changes in the gut microbiome over time [, 24, 25]. Likewise, NTS could be adopted for monitoring the ocular microbiome in real-time and even function as a prognostic tool for ocular infectious and inflammatory conditions when validated further [26].

However, as with any sequencing technique, it has its limitations in determining which organisms are merely colonizers or contaminants, rather than pathogenic organisms. In response to this issue, researchers have applied variable cutoff values (e.g.  > 20 mapped read pairs per million read pairs (rM) [27],  > 50 reads [28],  > 10 reads per million (RPM) ratio metric [29] and  > 500 reads [30]) to limit the over-interpretation of low abundance microorganisms. A recent study using single gene targeted nanopore sequencing provided evidence that the samples having < 20 reads generally had a low load of pathogen [31]. Similarly, in our study, the cut-off value for the positive diagnosis was 20 reads. Besides, we have segregated the pathogenic microorganisms from those that are known to be commensals (as shown in Tables 2 and 3). In summary, clinicians need to evaluate NTS results carefully and avoid antibiotic abuse.

## Conclusion

In conclusion, by comparing culture and NTS results, and analyzing patients’ clinical-oriented aspects, we demonstrated the superiority of NTS in diagnosing and guiding early treatment of endophthalmitis. Based on previous studies, we expanded the sample size to further elucidate the role of the NTS technique in clinical settings. NTS has already shown great potential for clinical applications due to its features of long-read sequences and real-time analysis. It promises to be an exceptionally powerful supplementary to traditional culture methods.

## Appendix A

See Table 6.

**Table 6:** Different sequencing methods and their positivity rates in suspected endophthalmitis

Team	Technique	Sample capacity	Positivity rate	References
Zhu (2022)	Culture mNGS	36	27.8% 88.9%	[32]
Low (2022)	Culture Illumina WGS 16S Nanopore Nanopore WGS	23 20 18 23	78.3% 73.9% 75% 83.3%	[33]
Huang (2021)	Culture NTS	18	44.4% 94.4%	[10]
Jun (2021)	Culture (AH) Culture (VH) NanoAmpli-Seq (AH) NanoAmpli-Seq (VH)	8	37.5% 75% 100% 75%	[18]
Mishra (2021)	NGS PCR	16	100% 62.5%	[34]
Selva Pandiyan (2020)	Culture Panbacterial PCR	88	19.3% 34.1%	[35]
Kosacki (2020)	Culture Panbacterial PCR Culture and PCR	142 137 128	54.2% 48.9% 64.1%	[36]
Deshmukh (2019)	Culture	34	44.1%	[37]
	Illumina NGS		88.2%	
Gandhi (2019)	Culture Illumina NGS	75	24% 86.7%	[38]
Mishra (2019)	Traditional Culture	195	8.7%	[39]
	Automated Culture Broad-range PCR		30.8% 65.1%	
Pongsachareonnont (2017)	Plate Culture Blood Culture PCR	41	12.2% 26.8% 26.8%	[40]
Lee (2015)	Culture qPCR Illumina NGS	21	66.7% 47.6% 57.1%	[41]
Bharathi (2013)	Culture PCR	66	24% 65%	[42]
Chiquet (2008)	Culture Eubacterial PCR Culture and PCR	100	38.2%; 54%/9% * 34.6%; 57%/70% 47%; 68%/72%	[43]
Chiquet (2007)	Culture Eubacterial PCR Culture and PCR	30	32% 61% 71%	[44]

**AH*: sample; *VH*: sample (before/after usage of antibiotics)
